# The relationship between family cohesion and adaptability and non-suicidal self-injury behavior in ethnic minority adolescents: a moderating mediation model

**DOI:** 10.3389/fpsyg.2023.1206889

**Published:** 2023-10-19

**Authors:** Junrong Lai, Zhiyan Chen

**Affiliations:** ^1^CAS Key Laboratory of Mental Health, Institute of Psychology, Chinese Academy of Sciences, Beijing, China; ^2^Department of Psychology, University of Chinese Academy of Sciences, Beijing, China

**Keywords:** non-suicidal self-injury, family cohesion and adaptability, depression, school connectedness, adolescents

## Abstract

To explore the relationship between family cohesion and adaptability and non-suicidal self-injury behavior among ethnic minority adolescents, as well as the mediating effect of depression and the moderating effect of school connectedness, this study adopts the Family Adaptability and Cohesion Scale, the Non-Suicidal Self-Injury Behavior Questionnaire, the Center for Epidemiological Studies Depression Scale, and the School Connectedness Scale to collect behavioral data from 949 ethnic minority middle-school students. Descriptive statistical analysis and correlation analysis, as well as the mediating and moderating effects, were performed using SPSS 25.0 and the PROCESS macro program. We found that family cohesion and adaptability significantly and negatively predicted non-suicidal self-injury in ethnic minority adolescents (*β* = −0.28, *p* < 0.001); depression mediated the relationship between family cohesion and adaptability and non-suicidal self-injury in minority adolescents, with a confidence interval (mediating effect size −0.15, and a Bootstrap 95% CI) of [−0.19, −0.12]. School connectedness moderated the second half of the mediating effect (*β* = −0.08, *p* < 0.01).

## 1. Introduction

Non-suicidal self-injury is defined as self-injury behavior that is intentionally caused to the body surface and is unrecognized by society as having been caused with suicidal intention (Brown and Plener, 2017). Long-term and repeated non-suicidal self-injury is related to depression, anxiety, marginal personality, post-traumatic stress, and other psychological disorders (Wen et al., 2020; Zhou and Jiang, 2021; Chen et al., 2022) and is also an important risk indicator for predicting suicide ideation and suicide attempts (Guan et al., 2012). Teenagers are at high risk of self-injuring behavior (Hankin et al., 2015). Previous research has shown that the rate of non-suicidal self-injury among adolescents is on the rise (Plener et al., 2015). The proportion of non-suicidal self-injury among adolescents in China is 36–57% (Jiang et al., 2011). Currently, the domestic research on non-suicidal self-injury of adolescents mainly focuses on middle school students of the Han nationality, while less attention is paid to ethnic minority adolescents living in remote and poor rural areas. Ethnic minority adolescents in this study are considered a group of people of a particular race or nationality living in China where most people are from a different race or nationality except the Han nationality. In the last 20 years, the mental health levels of ethnic minority adolescents in China have shown a downward trend. Therefore, it is important to explore the influencing factors and mechanisms of non-suicidal self-injury among ethnic minority adolescents and provide theoretical references and practical suggestions for psychological intervention for ethnic minority adolescents.

As one of the main environments for individual growth and socialization, family life has an important impact on the psychological development of teenagers (Chi and Xin, 2001). According to the theory of the family ring model, higher family cohesion and adaptability are conducive to the exertion of family functions (Fang et al., 2004), while families with poor cohesion and adaptability are prone to children suffering from psychosomatic diseases (Dou et al., 2023), behavior irregularities, and other maladjustments (Yi, 1998). Family cohesion and adaptability refer to the emotional connection between family members, the degree of autonomy experienced by individuals in the family system, and the ability of the family system to change its power structure, role relationships, and relationship rules to cope with situations and development pressures (Olson et al., 1979). Research shows that there is a significant positive correlation between family cohesion and adaptability and the mental health levels of junior high school students (Cao et al., 2010), that is, the higher the family cohesion and adaptability, the higher the mental health levels. Another study found that compared with individuals without non-suicidal self-injury, non-suicidal self-injury adolescents have lower levels of family cohesion and adaptability (Lin et al., 2020)^.^ Therefore, this study proposes hypothesis 1: family cohesion and adaptability significantly negatively predict non-suicidal self-injury among ethnic minority adolescents.

According to the self-system process model, external environmental resources, such as family cohesion and adaptability, will affect the development outcomes, such as non-suicidal self-injury, through the individual's internal psychological state, such as depression (Ryan and Deci, 2000). As a common emotional state in teenagers, depression is an emotional experience of pain, depression, and sadness caused by an individual's inability to cope with negative events in life (Thapar et al., 2012). According to the experience avoidance model, when family factors trigger individual negative emotions, they may resort to non-suicidal self-injury as a means of escaping unpleasant emotions so that the negative emotions can be alleviated (Wang et al., 2017). Previous research shows that adolescents with early emotional characteristics, such as depression and inferiority, are more likely to suffer from self-injury (Keenan et al., 2014). This implies that individuals with higher levels of depression are more likely to suffer from self-injury (O'Connor et al., 2010). Therefore, this study proposes hypothesis 2: depression plays a mediating role between family cohesion and adaptability and non-suicidal self-injury in ethnic minority adolescents.

Although family cohesion and adaptability may have an important impact on non-suicidal self-injury among ethnic minority adolescents in Yunnan through the indirect pathway of depression, there may be some individual differences in this effect. According to the motivation-volition model, most individuals with self-injury motivation will not continue to attempt self-injury behavior, among which high levels of social support, positive thinking about the future, and goal reinvestment are typical moderating factors (O'Connor et al., 2012).

The school is an important place to cultivate and form social relationships among young people and provides a suitable social environment for their physical and mental development (Nickerson et al., 2011). Adolescents spend more time in school activities, and social support provided by schools increases the chances of positive mental health development of adolescents (Zou et al., 2022). Studies have shown that students with higher levels of school connection are less likely to experience substance abuse, violent or deviant behavior, suicidal thoughts, or suicide attempts (Lonczak et al., 2002). On the contrary, if students do not experience a connection with their school, do not feel the care of their teachers, or feel that they have no friends at school and do not belong to the school, they will feel lonely and insecure, and they are more likely to use drugs and engage in violent behavior; they are also at greater risk of serious internalizing behaviors such as eating disorders and suicide attempts (Resnick et al., 1993). When students like their school, care about the teacher's opinion of them, respect the authority of the school, and connect with the school, they will actively internalize the school's goals and values, thereby reducing the possibility of negative behavior (Yin and Jia, 2014). Previous research on adolescents showed that school connection had a significant moderating effect between negative emotions and non-suicidal self-injury (Xiang et al., 2019). Additionally, another study showed that school connection had a significant effect on non-suicidal self-injury behavior (Wang et al., 2021).

**Figure 1:**
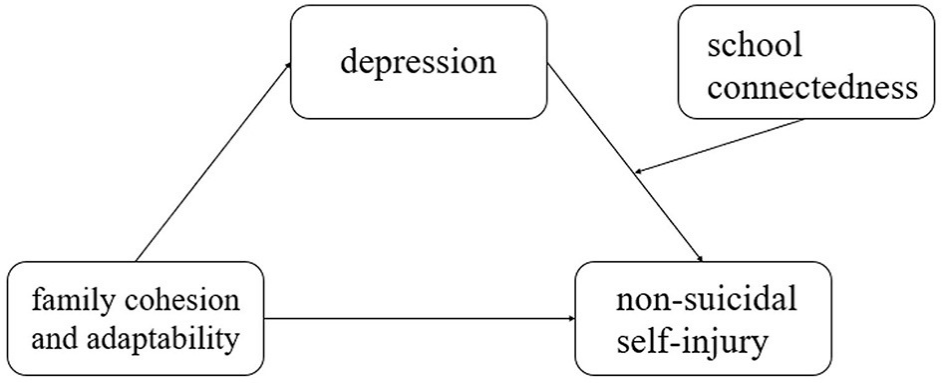
Hypothetical model of mediation of depression and regulation of school connection.

Based on the motivation-volition model and previous research, this study proposes hypothesis 3: school connection mediates the second half of the mediating effect of family cohesion and adaptation-depression non-suicidal self-injury among ethnic minority adolescents in Yunnan (Figure 1).

## 2. Methods

### 2.1. Participants

A total of 1,015 junior middle school students in Yunnan Province were selected as the research participants, and 949 valid questionnaires were recovered, with an effective recovery rate of 93.50%. All participants were from five junior middle schools. All participants and parents gave informed consent by telephone. Among them, 490 were boys (51.63%) and 459 were girls (48.37%). The average age of the participants was (13.86 ± 0.86) years old and the age range of participants was 10–15 years. More details on the general sociodemographic information of the participants are included in Supplementary Table S1. This study was approved and consented to by the Ethics Committee of the Institute of Psychology, Chinese Academy of Sciences.

### 2.2. Measures

#### 2.2.1. Family cohesion and adaptability scale

The Second Edition of the Family cohesion and adaptability Scale prepared by Olson and revised by Fei et al. (1991) was adopted, with 30 items in total. A 5-level score was adopted, ranging from 0 (never) to 5 (always). Higher scores indicate that individuals have higher levels of family cohesion and adaptability. In this study, the Cronbach's alpha coefficient of the scale is 0.91.

#### 2.2.2. Questionnaire on non-suicidal self-injuring behavior of adolescents

The non-suicidal self-injuring behavior assessment questionnaire for adolescents (Wan et al., 2018) was conducted to examine whether the adolescents had non-suicidal self-injuring behavior and its related frequency in the last year. It has 12 items in total, and a 5-level score was adopted, ranging from 0 (never) to 4 (always). The higher the score, the higher the degree of non-suicidal self-injury in the last year. There are two types of non-suicidal self-injuring behavior, namely, non-suicidal self-injuring behavior without obvious tissue damage and non-suicidal self-injuring behavior with obvious tissue damage. For example, one item is “Deliberately strangling myself.” It has good reliability and validity and can be used as an assessment tool for non-suicidal self-injury behavior and function in Chinese adolescents. In this study, the Cronbach's alpha coefficient of the questionnaire is 0.80.

#### 2.2.3. Epidemic center depression scale

The Chinese simplified version of the Epidemic Investigation Center Depression Scale revised by He Jin (He et al., 2016) was adopted. There were nine items in total. A 5-level score was adopted, ranging from 0 (little) to 3 (most or all of the time). The higher the score, the higher the frequency of depressive symptoms. In this study, the Cronbach's alpha coefficient of the scale is 0.82.

#### 2.2.4. School connectedness scale

The school connectedness scale prepared by Resnick et al. (1993) and revised by Yu et al. (2011) was used. There were 10 items in total, and 5 grades were used, ranging from 1 (completely disagree) to 5 (completely agree). The higher the score, the higher the individual's school connectedness. In this study, the Cronbach's alpha coefficient of the scale is 0.81.

### 2.3. Procedure

The study was reviewed and approved by the local ethics committee. Before the questionnaire was administered, the school's moral education teachers uniformly trained the head teachers of each class on the test administration process and precautions. The head teacher informed the parents of the test and the parents and students voluntarily signed the informed consent. During the test, the head teacher of each class uniformly organized the subjects to distribute paper questionnaires to answer during the class meeting time. All the subjects completed all the questionnaires within approximately 40 min. After the test was completed, the head teacher took the questionnaires back and kept them confidential.

### 2.4. Data processing

A unified questionnaire was used for the test. The participants were instructed to answer carefully according to the actual situation, and the confidentiality of personal information was emphasized. SPSS25.0 was used to input and manage the collected data, and descriptive statistical analysis and correlation analysis were carried out. After standardizing scores for each scale, the two models were tested in the PROCESS macro program (Hayes, 2013). Model 4 was used to test the mediating effect of depression, and Model 14 was used to test the moderating effect of school bonding. After Bonferroni correction, the threshold value of *P* < 0.05 was considered statistically significant.

## 3. Result

### 3.1. Control and inspection of common method bias

Since data were collected using a self-reported method, the results may be affected by common method bias, so the Harman single-factor method was used to test the common method bias, and 13 factors with eigenvalues >1 were obtained, accounting for 54.82% of the variance. The variance explained by the first factor was 20.33%, less than the critical value of 40%. Therefore, there is no serious common method bias in this study.

### 3.2. Average, standard deviation, and correlation matrix of each variable

The results of description and correlation analysis showed that (see Table 1) family cohesion and adaptability were significantly negatively correlated with non-suicidal self-injury and depression and significantly positively correlated with school connectedness. There was a significant positive correlation between non-suicidal self-injury and depression, and a significant negative correlation between non-suicidal self-injury and school connectedness. There was a significant negative correlation between depression and school connectedness. Gender and age were significantly correlated with the main research variables, and they were treated as control variables in the subsequent analysis.

**Table 1:** Descriptive statistics and correlation analysis results of each variable (*n* = 949).

	***M***	***SD***	**1**	**2**	**3**	**4**	**5**	**6**
1. Gender	1.48	0.5	1					
2. Age	13.86	0.86	−0.14^***^	1				
3. Family cohesion and adaptability	96.66	19.99	−0.08^**^	−0.04	1			
4. Non-suicidal self-injury	1.87	3.13	0.12^***^	−0.02	−0.29^***^	1		
5. Depression	7.52	5.04	0.12^***^	0.13^***^	−0.42^***^	0.43^***^	1	
6. School connectedness	35.78	6.77	−0.03	−0.11^***^	0.46^***^	−0.32^***^	−0.50^***^	1

^**^P < 0.01, ^***^P < 0.001.

### 3.3. The relationship between family cohesion and adaptability and non-suicidal self-injury: a moderating mediation model

First, Model 4 in the PROCESS macro was used to test the mediating effect of depression in the relationship between family cohesion and adaptability and non-suicidal self-injury under the condition of controlling gender and age. All variables except demographic variables have been standardized. As shown in Table 2, family cohesion and adaptability negatively predicted non-suicidal self-injury (β = −0.28, *p* < 0.001) and negatively predicted depression (β = −0.40, *p* < 0.001). When family cohesion and adaptability and depression predicted non-suicidal self-injury at the same time, family cohesion and adaptability could still significantly negatively predict non-suicidal self-injury (β = −0.13, *p* < 0.001), depression significantly positively predicted non-suicidal self-injury (β = 0.38, *p* < 0.001). The bootstrap method based on deviation correction percentile further found that depression played a partial mediating role between family cohesion and adaptability and non-suicidal self-injury, and its 95% confidence interval was [−0.19, −0.12]. The mediating effect (−0.15) accounted for 53.57% of the total effect (−0.28).

**Table 2:** The test of mediation model of depression.

**Regression equation**		**Overall fitting index**			**Significance of regression coefficient**	
**Outcome variable**	**Predictive variables**	***R***	***R** ^2^*	***F***	β	***t***
Non-suicidal self-injury	Gender	*0.30*	*0.09*	32.11^***^	0.20	3.12^**^
	Age				−0.02	−0.57
	Family cohesion and adaptability				−0.28	−8.96^***^
Depression	Gender	0.44	0.20	76.96^***^	0.21	3.56^***^
	Age				0.15	4.42^***^
	Family cohesion and adaptability				−0.40	−13.71^***^
Non-suicidal self-injury	Gender	0.45	0.21	61.23^***^	0.12	1.98^*^
	Age				−0.08	−2.25^*^
	Family cohesion and adaptability				−0.13	−4.01^***^
	Depression				0.38	11.62^***^

^*^P < 0.05, ^**^p < 0.01, ^***^p < 0.001.

Secondly, Model14 in the PROCESS macro was used to test the moderation effect of school connectedness under the condition of controlling for gender and age. All variables except demographic variables have been standardized. The results are shown in Table 3. After joining the school connectedness, the interaction term of depression and school connectedness significantly negatively predicted non-suicidal self-injury (β = −0.08, *p* < 0.01). To sum up, family cohesion and adaptability, depression, school connectedness, and non-suicidal self-injury constitute a moderated model with mediation. Specifically, school connection moderated the second half of the path of non-suicidal self-injury of ethnic minority adolescents through depression. Additionally, the moderating role of school connectedness in the first part of mediation has been conducted, but there were no significant results about this moderating role.

**Table 3:** The test of mediating moderating effect.

**Regression equation**		**Overall fitting index**			**Significance of regression coefficient**			
**Outcome variable**	**Predictor Variable**	***R***	***R** ^2^*	***F***	β	***Bootstrap lower limit***	***Bootstrap upper limit***	***t***
Depression	Gender	0.44	0.20	76.96^***^	0.21	0.09	0.33	3.56^***^
	Age				0.15	0.08	0.22	4.42^***^
	Family cohesion and adaptability				−0.40	−0.46	−0.34	−13.71^***^
Non-suicidal Self-injury	Gender	0.47	0.22	44.65^***^	0.12	0.01	0.24	2.07^*^
	Age				−0.09	−0.15	−0.02	−2.52^*^
	Family cohesion and adaptability				−0.10	−0.16	−0.03	−2.95^**^
	Depression				0.32	0.25	0.39	9.03^***^
	School connectedness				−0.11	−0.18	−0.04	−3.12^**^
	Depression × school connectedness				−0.08	−0.14	−0.03	−2.91^**^

^*^P < 0.05, ^**^p < 0.01, ^***^p < 0.001.

**Figure 2:**
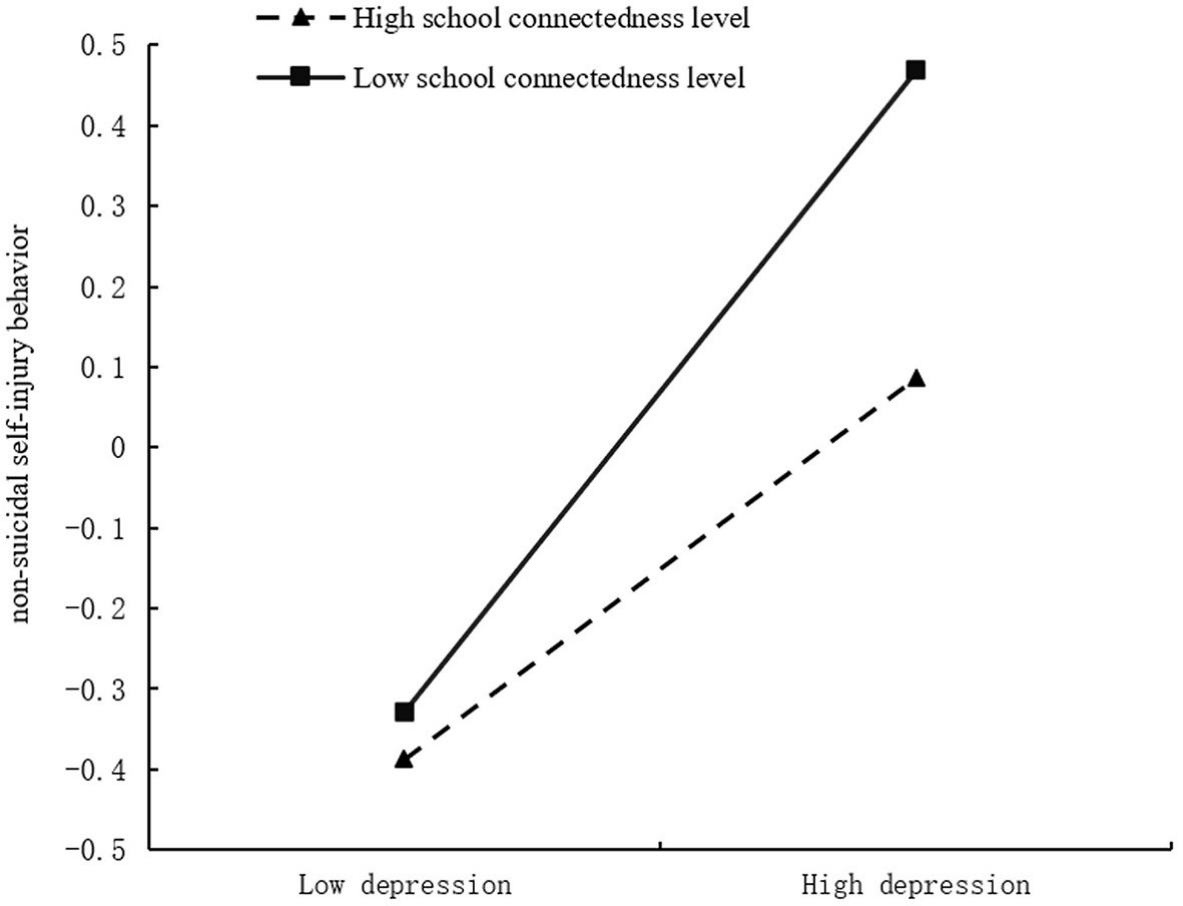
The moderating effect of school connectedness.

In order to better explain the moderating effect, a simple slope analysis was performed. It can be seen from Figure 2 that when the individual's school connectedness level was low (*M*- 1*SD*), depression significantly positively predicted the non-suicidal self-injury of ethnic minority adolescents (βsimple = 0.40, *t* = 9.67, *p* < 0.001). When the individual's level of school connectedness was high (*M* + *1SD*), depression still positively predicted non-suicidal self-injury of ethnic minority adolescents, but its predictive effect was reduced (βsimple = 0.24, *t* = 4.90, *p* < 0.001). The results showed that with the improvement of school connectedness, the predictive effect of depression on non-suicidal self-injury of ethnic minority adolescents decreased.

## 4. Discussion

### 4.1. The relationship between family cohesion and adaptability and non-suicidal self-injury

This study found that family cohesion and adaptability significantly negatively predicted the non-suicidal self-injury of ethnic minority adolescents, which is consistent with previous research (Hu et al., 2019; Su et al., 2021). This shows that good family cohesion and adaptability were important protective factors for non-suicidal self-injury in adolescents and provided good environmental conditions for the development of the mental health of adolescents. According to the Circumplex model of marital and family systems, when family members' intimacy and adaptability are unbalanced, such as when family members are overly interventionist or completely indifferent to each other's lives, children will lack attachment and respect for their parents, which leads to emotional problems such as maladjustment to the surrounding environment and being prone to self-injuring thoughts and behaviors (Olson, 2000). Therefore, it is necessary to pay attention to the family environment of ethnic minority adolescents and strengthen the local mental health work related to family education (Fu and Zhang, 2021).

### 4.2. The mediating role of depression

This study also found that family cohesion and adaptability affect non-suicidal self-injury in ethnic minority adolescents through depression. When individuals have families with low intimacy and poor adaptability for a long time and lack emotional communication and support from their parents, the family atmosphere will gradually become inharmonious, making the individual feel lonely, depressed, withdrawn, and silent, which leads to depression (Yang, 2001). On the other hand, according to the experience avoidance model, individuals who hurt themselves often fail to regulate negative emotions in an adaptive way, so individuals tend to use self-injury to avoid or alleviate unpleasant emotional experiences (Feng, 2022). Previous studies have shown that relieving negative emotions is the main reason for self-injuring behavior. When self-injury occurs, negative emotions will also be significantly relieved (Lin, 2022). Therefore, ethnic minority adolescents are more likely to develop negative emotions such as depression, when faced with stress or stimuli from their families. Individuals use non-adaptive and inappropriate coping strategies, such as self-injury, to alleviate negative emotions, which may increase the possibility of self-injury. This reminds school mental health workers and relevant departments that when the family cohesion and adaptability of ethnic minority adolescents cannot be improved in a short period of time, they can reduce the levels of depression in adolescents through appropriate psychological counseling and psychological intervention in order to reduce non-suicidal self-injuring behaviors, for example, teaching them more skills and methods to prevent and alleviate emotional problems, such as cognitive reappraisal and interpersonal support, and how to use these methods to solve difficulties in daily learning and life (Fu and Zhang, 2021).

### 4.3. Moderating effects of school connectedness

This study also found that school connection mediates the relationship between depression and non-suicidal self-injury among ethnic minority adolescents in Yunnan, that is, family intimacy and the second half of the mediating effect of adaptation-depression non-suicidal self-injury. When ethnic minority adolescents feel low family intimacy and adaptability, they are prone to depression, but when they feel a strong school connection, non-suicidal self-injury behaviors caused by depression can be reduced; thus, school connection can intervene in the mental health of ethnic minorities in Yunnan. The process of transformation of depression caused by low family intimacy and adjustment into non-suicidal self-injury among adolescents in ethnic minority areas. School connection is regarded as a protective factor in reducing adolescents' susceptibility to health risks and participation in abnormal behaviors (Dornbusch et al., 2001), and when students have a stronger connection with school, it can reduce emotional distress and suicidal ideation (Resnick, 1997). According to the motivation-volition model, although adolescents from ethnic minorities experience family intimacy and poor adaptability and experience negative emotions such as depression, when they feel the care of teachers and classmates, they will feel that they are members of the school. One molecule produces a higher sense of belonging and happiness, alleviates the process of repeated exposure to pain, and alleviates the occurrence of self-injury behavior to a certain extent (McNeely et al., 2002). Therefore, we need to fully consider the social living environments of adolescents in ethnic minority areas in Yunnan, especially the school environment, and pay attention to the interaction between teachers and students, and between students and students, in order to formulate effective psychological intervention and preventive measures (Burns et al., 2015). For example, schools can strengthen teachers' knowledge surrounding adolescent psychology, treat students with care and fairness, and actively guide students to participate in learning in order to improve support for students and promote students' higher school connection (McNeely et al., 2002) by increasing skills training; encouraging teachers to adopt interactive and cooperative learning methods; setting courses on problem-solving, self-management, and self-control skills; and reducing inappropriate ways of solving problems.

### 4.4. Limitations

Firstly, this study adopts a cross-sectional design, and it cannot reveal the causal relationship between variables like a longitudinal study, but through an independent cross-sectional sampling study of ethnic minorities, it can improve the overall non-suicidal self-injury rate of adolescents under the condition of avoiding the loss of subjects. In the future, it is necessary to assess the question of intra-individual change over time, and longitudinal research can be used for further verification. Secondly, the data used in this study was self-reported by the students and, therefore, might have a social approval effect. Future research could comprehensively use teacher assessment, parent assessment, and other methods to collect data. Finally, the participants of this study were minority students. Whether the research results could be extended to other groups needs to be further tested. Future research could consider adding more groups (such as the Han nationality) to test and compare results.

## 5. Conclusions

A total of 949 minority junior high school students participated in the study, SPSS software and PROCESS macro program were used to test the sample data, and the sample distribution was reconstructed through the random sampling of the original samples (a total of 5,000 samples were constructed in this study, each with a sample size of 949 people), and the robust standard error and confidence interval of parameter estimations were obtained. The results showed that family cohesion and adaptability influence non-suicidal self-injury in ethnic minority adolescents through depression, and the second half of the mediating effect was moderated by school connectedness. According to the above conclusions, some suggestions and relevant educational and teaching measures were put forward for psychological intervention in ethnic minority adolescents.

## Data availability statement

The raw data supporting the conclusions of this article will be made available by the authors, without undue reservation.

## Ethics statement

The studies involving humans were approved by Institute of Psychology, Chinese Academy of Sciences. The studies were conducted in accordance with the local legislation and institutional requirements. The participants provided their written informed consent to participate in this study.

## Author contributions

JL: conceptualization, methodology, data curation, formal analysis, software, visualization, investigation, supervision, writing—original draft, and writing—review and editing. ZC: funding acquisition, project administration, supervision, and writing—review and editing. All authors contributed to the article and approved the submitted version.
