# Supplementary material for: The relationship between family cohesion and adaptability and non-suicidal self-injury behavior in ethnic minority adolescents: a moderating mediation model
Source: Front Psychol. 2023 Oct 19;14:1206889. doi: 10.3389/fpsyg.2023.1206889 (PMC10625406; doi:10.3389/fpsyg.2023.1206889)
Supplement: Supplementary file 1 [file Table_1.DOCX]

| Table S1 General sociodemographic information(n=949) | | | |
| --- | --- | --- | --- |
| Variables | Groups | Number of the participants | Percent (%) |
| Sex | Boy | 490 | 51.63 |
|  | Girl | 459 | 48.37 |
| Grades | Grade 1 | 467 | 49.2 |
|  | Grade 2 | 482 | 50.8 |
| Rural household registration | Yes | 842 | 88.72 |
|  | No | 107 | 11.28 |
| Only-child | Yes | 132 | 13.91 |
|  | No | 817 | 86.09 |
| Two-parent family | Yes | 793 | 83.56 |
|  | No | 156 | 16.44 |
| live on campus | Yes | 925 | 97.47 |
|  | No | 24 | 2.53 |
| Parents working outside the home | No | 406 | 42.78 |
|  | Father working outside | 170 | 17.91 |
|  | Mother working outside | 87 | 9.17 |
|  | Both working outside | 286 | 30.14 |
| Education level of father | Never went to school | 10 | 1.05 |
|  | Primary school | 311 | 32.77 |
|  | Junior high school | 478 | 50.37 |
|  | Senior high school | 112 | 11.80 |
|  | Junior college | 21 | 2.21 |
|  | Undergraduate college | 15 | 1.58 |
|  | Postgraduate or above | 2 | 0.21 |
| Education level of mother | Never went to school | 75 | 7.90 |
|  | Primary school | 445 | 46.89 |
|  | Junior high school | 331 | 34.88 |
|  | Senior high school | 77 | 8.11 |
|  | Junior college | 11 | 1.16 |
|  | Undergraduate college | 9 | 0.95 |
|  | Postgraduate or above | 1 | 0.11 |
